# The Janus face of HMGB1 in heart disease: a necessary update

**DOI:** 10.1007/s00018-018-2930-9

**Published:** 2018-10-10

**Authors:** Angela Raucci, Stefania Di Maggio, Francesco Scavello, Alessandro D’Ambrosio, Marco E. Bianchi, Maurizio C. Capogrossi

**Affiliations:** 1 Unit of Experimental Cardio-Oncology and Cardiovascular Aging, Centro Cardiologico Monzino-IRCCS, Via C. Parea 4, 20138 Milan, Italy; 2 Chromatin Dynamics Unit, Università Vita-Salute San Raffaele, Milan, Italy; 3 Department of Cardiology, Ochsner Medical Center, New Orleans, USA; 4 Present Address: Department of Experimental Oncology, European Institute of Oncology, Milan, Italy; 5 Present Address: Division of Cardiology, Johns Hopkins Bayview Medical Center, Baltimore, USA

**Keywords:** Alarmin, Regeneration, Inflammation, Oxidative stress, Biomarker

## Abstract

High mobility group box 1 (HMGB1) is a ubiquitous nuclear protein involved in transcription regulation, DNA replication and repair and nucleosome assembly. HMGB1 is passively released by necrotic tissues or actively secreted by stressed cells. Extracellular HMGB1 acts as a damage-associated molecular pattern (DAMPs) molecule and gives rise to several redox forms that by binding to different receptors and interactors promote a variety of cellular responses, including tissue inflammation or regeneration. Inhibition of extracellular HMGB1 in experimental models of myocardial ischemia/reperfusion injury, myocarditis, cardiomyopathies induced by mechanical stress, diabetes, bacterial infection or chemotherapeutic drugs reduces inflammation and is protective. In contrast, administration of HMGB1 after myocardial infarction induced by permanent coronary artery ligation ameliorates cardiac performance by promoting tissue regeneration. HMGB1 decreases contractility and induces hypertrophy and apoptosis in cardiomyocytes, stimulates cardiac fibroblast activities, and promotes cardiac stem cell proliferation and differentiation. Interestingly, maintenance of appropriate nuclear HMGB1 levels protects cardiomyocytes from apoptosis by preventing DNA oxidative stress, and mice with HMGB1cardiomyocyte-specific overexpression are partially protected from cardiac damage. Finally, higher levels of circulating HMGB1 are associated to human heart diseases. Hence, during cardiac injury, HMGB1 elicits both harmful and beneficial responses that may in part depend on the generation and stability of the diverse redox forms, whose specific functions in this context remain mostly unexplored. This review summarizes recent findings on HMGB1 biology and heart dysfunctions and discusses the therapeutic potential of modulating its expression, localization, and oxidative-dependent activities.

## Introduction

Cardiac diseases remain a leading cause of morbidity and mortality worldwide [1]. The adult heart is an organ with limited regenerative potential because of the low ability of cardiomyocytes (CMs) to proliferate after injury [2, 3]. The inflammatory response following cardiac damage consists of recruitment of immune cells that, in turn, guide the production of regenerative and healing mediators [4]. Cardiac repair occurs through the process of remodeling, involving mainly scarring orchestrated by cardiac fibroblasts (CFs), inflammatory cells and cardiomyocyte hypertrophy [4]. Strategies to enhance healing or prevent degeneration of a damaged heart are clinically relevant and represent an active research area.

High mobility group box 1 (HMGB1) is an architectural non-histone chromatin-binding protein regulating transcription, DNA replication and repair, and nucleosome structure and number [5–7]. Cells lacking HMGB1 contain 20% fewer nucleosomes and 30% more RNA transcripts genome-wide, and have an increased susceptibility to DNA damage [6]. In addition to its nuclear role, HMGB1 functions as an extracellular “alarmin” [8]. When cells die after trauma or infection, HMGB1 is passively released in the extracellular milieu, signals danger to the surrounding cells, activates innate and adaptive immunity and eventually promotes tissue repair [9, 10]. HMGB1 can also be secreted by activated cells after relocation from the nucleus to the cytosol [11]. Exogenous HMGB1 activity depends on the protein redox state and on several receptors including the receptor for advanced glycation endproducts (RAGE), Toll-like receptor 2 and 4 (TLR2-4) and C-X-C-chemokine receptor 4 (CXCR4) [12, 13]. Noteworthy, the dual location of HMGB1 may be functionally complementary, because HMGB1 secretion entails HMGB1 depletion in the nucleus [14]. As a major mediator of acute and chronic inflammation, extracellular HMGB1 plays a role in a variety of diseases and represents a promising pharmacologic target in multiple pathologic conditions [15].

In this review, we focus on the recent findings on HMGB1 redox functions and its role in cardiac dysfunctions.

## HMGB1: a multifunctional redox-sensitive protein

### Structure and post-translational modifications of HMGB1

HMGB1 is an evolutionary conserved chromatin-binding factor present in the nucleus of almost every cell type [16]. HMGB1 is essential for proper development since *Hmgb1*^−*/*−^ mice die soon after birth [17]; however, inducible whole body *Hmgb1*^−*/*−^ mice survive during adult life [18]. It belongs to the HMGB family and in mammals has three paralogs, HMGB2, HMGB3 and HMGB4; HMGB family members share the ability to bind DNA without sequence specificity and induce conformational and structural changes [5].

**Fig. 1:**
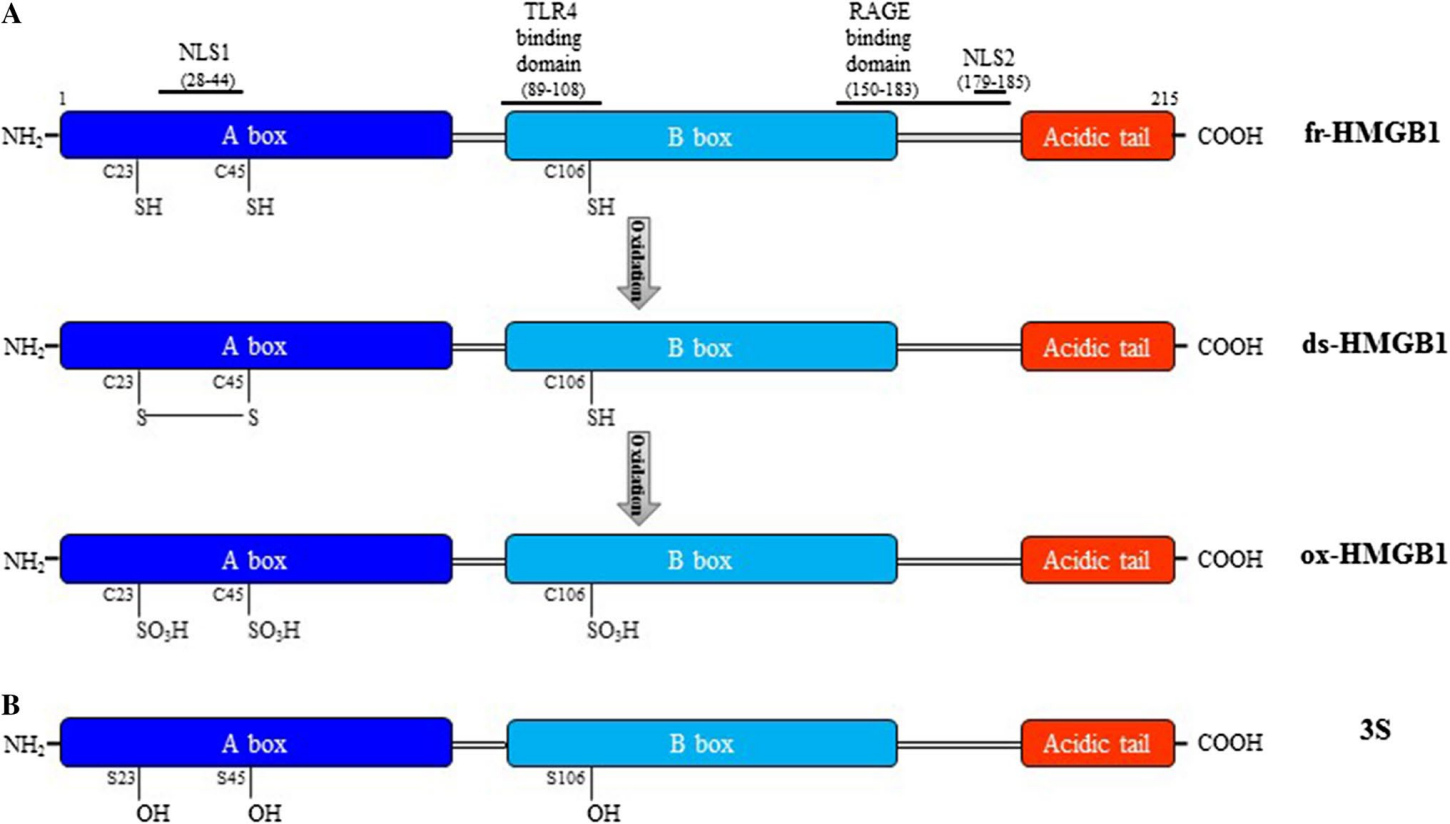
Structure and redox modifications of HMGB1. **a** HMGB1 comprises two DNA-binding domains, named A and B box, and a C-terminal acid tail connected by linker regions. HMGB1 has two lysine-rich nuclear localization sequences, NLS1 and NLS2, localized in the A box and in the linker region between the B box and the acidic tail, respectively. The domains recognized by TLR4 and RAGE are depicted. Three redox forms of HMGB1 depend on the redox conditions of the environment. The intracellular fully reduced HMGB1 (fr-HMGB1) with the three conserved cysteines in the reduced thiol state can be oxidized in the extracellular space to disulfide HMGB1 (ds-HMGB1), characterized by a disulfide bond between C23 and C45, and a thiol C106, that after further oxidation can give rise to the sulfonyl HMGB1 (ox-HMGB1) with cysteines carrying the sulfonyl group. **b** The non-oxidizable HMGB1 3S mutant. Recombinant 3S has been generated by substitution of cysteines with serine residues (S23–S45–S106)

Human HMGB1 protein is composed of 215 amino acids organized in two DNA-binding domains, named A and B boxes, and a negatively charged C-terminal tail [19] (Fig. 1a). HMGB1 has two nuclear localization signals (NLS1 and NLS2) and two nuclear export signals (NESs) that imply the continuous shuttling of the protein between nucleus and cytoplasm; however, in physiological conditions, the nuclear concentration is higher than in the cytosol [11]. Post-translational modifications direct nucleus-cytoplasm shuttling of the protein in stressed/activated cells. NLS1 and NLS2 lysines acetylation by P300/CBP-associated factor (PCAF), CREB binding protein (CBP), and Histone acetyltransferase p300 (p300) reduce HMGB1 binding to the nuclear importin protein karyopherin-1, facilitating translocation to the cytosol [11]. Conversely, Sirtuin 1 (SIRT1) deacetylases HMGB1 promoting nuclear retention [20]. Translocation of HMGB1 is controlled also via serine/threonine phosphorylation catalyzed by the calcium/calmodulin-dependent protein kinase (CaMK) IV or the classical protein kinase C (cPKC) [21, 22], and monomethylation of lysine-42 in neutrophils [23].

HMGB1 has three conserved cysteines in position 23 and 45 in the A box, which can form a disulfide bond, and in position 106 in the B box (Fig. 1a). Those cysteines are susceptible to oxidation that affects the extracellular activities of the protein [13, 24].

### HMGB1 is the archetype of DAMPs

Inflammation is the first line of defense against pathogens or trauma, which are detected as pathogen-associated molecular pattern (PAMPs) and damage-associated molecular pattern (DAMPs) molecules, respectively [25]. PAMPs are microbial molecules carrying conserved molecular motifs that can activate cells of the innate and adaptive immunity [25, 26] after recognition by pattern-recognition receptors (PRRs), such as TLRs, RIG-I-like receptors (RLRs) and NOD-like receptors (NLRs) [26]. DAMPs are endogenous molecules with specific intracellular functions that are passively released by necrotic cells or actively secreted or exposed by stressed living cells after sterile injuries or infection; DAMPs, also named “alarmins”, are recognized by PRRs and signal danger, activate inflammation and eventually tissue repair [25]. Different DAMPs, including Adenosine Triphosphate (ATP), uric acid, Deoxyribonucleic acid (DNA), Ribonucleic acid (RNA), Interleukin-1α (IL1α), heat shock proteins (HSPs) and HMGB1, have been identified [10].

**Fig. 2:**
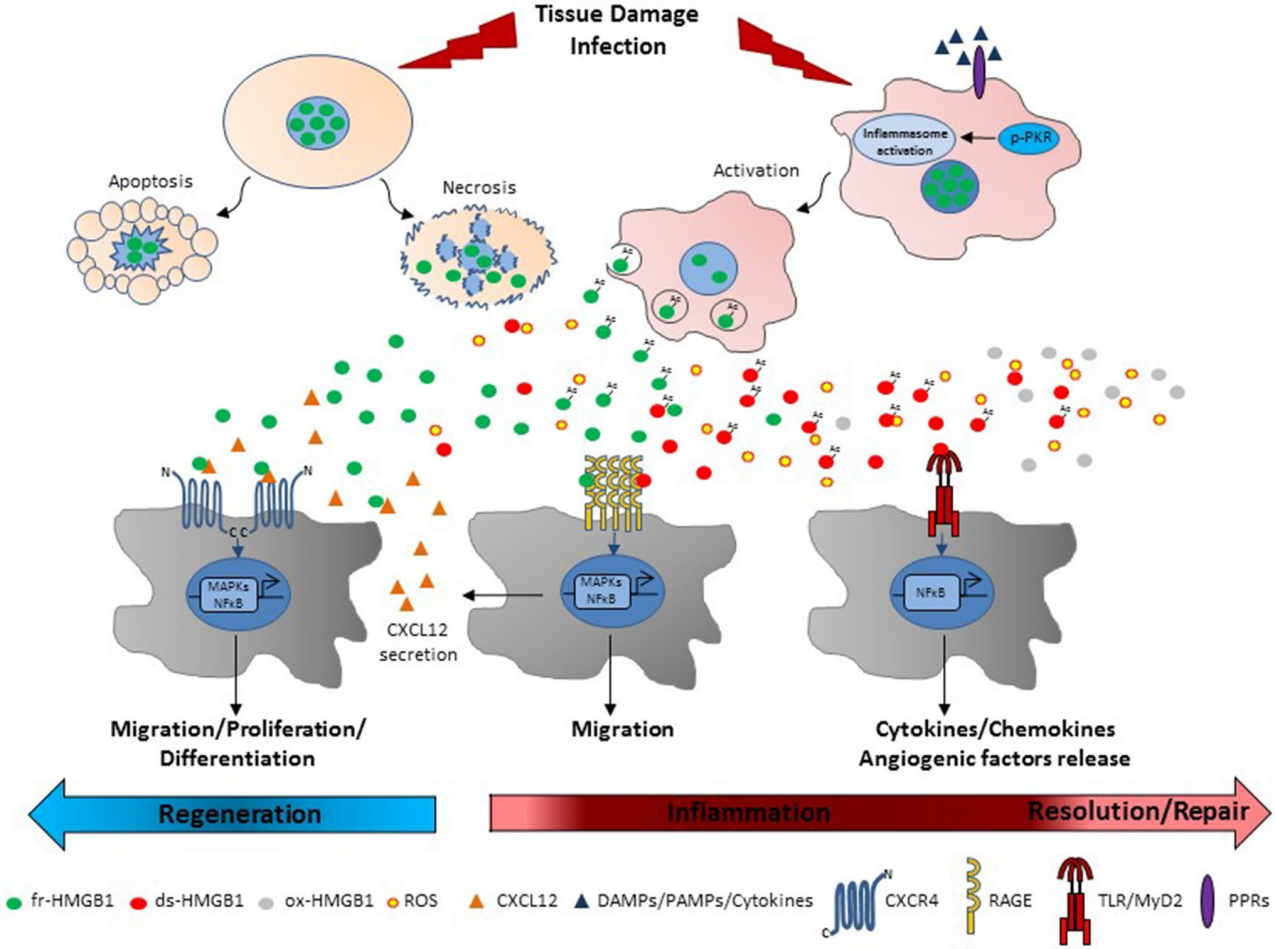
Extracellular functions of HMGB1 redox forms. After tissue damage or infection, non-acetylated fr-HMGB1 leaks out from necrotic cells. Acetylated (Ac) fr-HMGB1 is actively secreted by local immunocompetent and infiltrating immune cells upon inflammasome activation by PAMPs, DAMPs or pro-inflammatory stimuli. On the contrary, apoptotic chromatin tightly retains HMGB1. Whether acetylation of HMGB1 affects extracellular activity of HMGB1 is still unknown. Fr-HMGB1 interacts with CXCL12 to activate CXCR4-mediated cell migration, proliferation and differentiation to promote tissue healing and regeneration. HMGB1 also binds to RAGE to induce further production of CXCL12 and migration. MAPKs and NF-κB pathways are involved in these processes. In presence of reactive oxygen species (ROS), fr-HMGB1 is partially oxidized to ds-HMGB1 that binds to the TLR4-MD2 complex to stimulate the release of inflammatory and angiogenic factors through the activation of NF-κB. Further oxidation of ds-HMGB1 to sulfonyl ox-HMGB1 is associated mainly with the resolution of inflammation

HMGB1 is mainly expressed in the nucleus regulating transcription, replication, DNA repair and nucleosome assembly [5–7]. Further, HMGB1 is rapidly released by damaged cells [27, 28] and actively secreted by immune cells [11]. In contrast, HMGB1 is retained by the condensed chromatin of apoptotic cells (Fig. 2) [28]. Unlike other proinflammatory cytokines, secreted HMGB1 is a delayed mediator of inflammation that is released late via nonclassical secretion pathways [29, 30]. Other danger signals or proinflammatory stimuli induce HMGB1 hyperacetylation and nuclear translocation [31] along with double-stranded RNA dependent kinase (PKR) autophosphorylation [32]. Activated PKR interacts with inflammasome components, like NACHT, LRR and PYD domains-containing protein 3 (NLRP3), and promotes inflammasome activation that eventually drives HMGB1 release via pyroptosis [32]. Thus, active secreted HMGB1 is hyperacetylated (Fig. 2).

Extracellular HMGB1 activates innate and acquired immunity, promotes tissue repair, and regeneration [9, 10, 33]. HMGB1 directly affects fibroblasts, monocytes/macrophages, dendritic and endothelial cells activation and migration [34–37]. Inhibition of extracellular HMGB1 attenuates inflammation and confers protection in several animal models of experimental diseases including sepsis [30], cardiac and liver ischemia/reperfusion injury [38, 39], diabetes [40], autoimmune diseases [41] and epilepsy [42]. Several inhibitors have been identified and developed to counteract HMGB1 (Table 1; reviewed in [15]). It is noteworthy that, in a variety of tissues exogenous HMGB1 can support regeneration [18, 43–46], wound-healing [47] and reparative angiogenesis [33] by inducing stem cell priming, proliferation, migration and differentiation, and by recruiting healing macrophages to the damaged tissue [18, 44, 45, 48–50].

**Table 1:** Most known and used inhibitors of HMGB1

Inhibitor identification	Category	Way of action
Polyclonal antibody	–	Neutralizes HMGB1 action
2G7	Monoclonal Ab against aa 53–63 of HMGB1	Neutralizes HMGB1 action
MAb	Monoclonal Ab against aa 205–210 of HMGB1	Neutralizes HMGB1 action
DPH1.1	Monoclonal Ab against 17-mer peptide at the end of B box of HMGB1	Neutralizes HMGB1 action
Recombinant BoxA	Fragment of HMGB1 (2–89 aa)	Antagonizes fr-HMGB1 chemotactic activity; antagonist of CXCR4-antagonizes CXCL12 and 3S
Recombinant soluble receptor for advanced glycation endproducts (sRAGE)	Soluble receptor	Direct binding with ds-HMGB1 and fr-HMGB1
Ethyl pyruvate, ethacrynic acid	Anti-inflammatory small organic molecules	Inhibitors of HMGB1 nucleus-cytoplasm translocation and secretion
Glycyrrhizin (Gly) and derivates	Anti-inflammatory small organic molecules	Direct binding with fr-HMGB1
Salicylic acid (SA)	Anti-inflammatory small organic molecules	Direct binding with ds-HMGB1 and fr-HMGB1
P5779	Small synthetic peptide	Inhibits ds-HMGB1/MD-2 interaction

Hence, HMGB1 is a DAMP that can elicit both harmful and beneficial responses after tissue damage. This pleiotropic activity depends on HMGB1 sensitivity to the environmental oxidizing conditions that induce complex redox post-translation modifications.

### HMGB1 redox state coordinates its extracellular activities through different receptors

Based on the redox state of the cysteines, three redox forms of HMGB1 have been identified: fully reduced HMGB1 (fr-HMGB1) in which all cysteines are reduced, disulfide HMGB1 (ds-HMGB1) in which C23 and C45 are partially oxidized forming a disulfide bond, while the unpaired C106 is reduced and sulfonyl HMGB1 (ox-HMGB1) in which all cysteines are oxidized [13] (Fig. 1a). Supernatants of lipopolysaccharide (LPS)-activated monocytes contain both acetylated fr-HMGB1 and ds-HMGB1 [13]. In a murine model of acute muscle injury, endogenous HMGB1 released by necrotic cells is in the fully reduced state and it turns very soon in the disulfide form because of the oxidizing conditions of the extracellular space [13]. Eventually, ds-HMGB1 can be converted to the functionally inert ox-HMGB1 (Fig. 1a). Notably, fr-HMGB1 and ds-HMGB1 possess mutually exclusive activities [13]. Fr-HMGB1 exerts chemotactic action and skews polarization of macrophages toward a regenerative phenotype [13, 45]. Ds-HMGB1 stimulates pro-inflammatory cytokine/chemokine production in immune cells and is pro-angiogenic in endothelial cells [13, 24, 51, 52] (Fig. 2). A mutant of HMGB1, named 3S (Fig. 1b), in which cysteines have been substituted by serines, mimics fr-HMGB1 actions, likely because it is resistant to oxidation and cannot be converted to ds-HMGB1 or be inactivated by sulfonylation [13, 45, 53]. Ox-HMGB1 can be found in the late stage of the inflammatory process and is associated with the resolution/regenerative phase [24], and may influence the activation state of neutrophils [54].

The multifunctional activities of HMGB1 rely on the ability of the redox forms to bind different receptors, alone or in heterocomplex with others ligands [12]. The receptors most widely studied are RAGE, TLR2 and -4 and CXCR4 [12]. RAGE is a transmembrane receptor with structural features of adhesion molecules that recognizes several other proteins, i.e., advanced glycation endproducts (AGEs), S100/calgranulin proteins, amyloid β-peptides and extracellular matrix components [55, 56]. RAGE engagement signals through Mitogen-activated protein kinases (MAPKs) and nuclear factor kappa-light-chain-enhancer of activated B cells (NF-κB), inducing cell activation, proliferation and migration [57]. RAGE is involved in a variety of pathologies mediated by HMGB1, and this axis represents an important potential target [58]. RAGE regulates HMGB1-induced cell adhesion and migration [59–61]. Although all redox forms of HMGB1 interact with RAGE, ds-HMGB1 binds with higher affinity [45, 62]. Interaction between RAGE and fr-HMGB1 increases the transcription of the chemotactic gene stromal derived factor 1 (SDF-1) or C–X–C motif chemokine 12 (CXCL12) [13]. RAGE/ds-HMGB1 binding is necessary for platelet-dependent neutrophil activation and neutrophil extracellular traps (NETs) formation in thrombo-inflammatory lesions [62].

TLRs constitute a family of transmembrane molecules involved in host defense that have similar structure but differ in their subcellular localization and ligands [63]. TLR2 and TLR4 interact with HMGB1 leading to nuclear translocation of NF-κB and expression of pro-inflammatory cytokines in neutrophils and macrophages [64]. The TLR2/HMGB1 axis promotes natural killer (NK) and cancer stem cell activation [65, 66]. The HMGB1/nucleosome complex activates immune cells through TLR2 [67]. HMGB1/TLR4 or/TLR2 axes contribute to regulate inflammation during lung and liver injury, epilepsy, cancer, and heart disease [42, 68–72]. It is not known which HMGB1 redox form binds to TLR2 and whether binding is direct or mediated by other TLR2 ligands. Ds-HMGB1 stimulates cytokine/chemokine production in inflammatory cells through binding to TLR4 [24, 73] via formation of a complex with CD14 and TLR4 adaptor myeloid differentiation factor 2 (MD-2) [73–75] (Fig. 2). Prevention of ds-HMGB1/MD-2 interaction abrogates cytokine induction and protects against liver injury, chemical toxemia and sepsis [73].

**Fig. 3:**
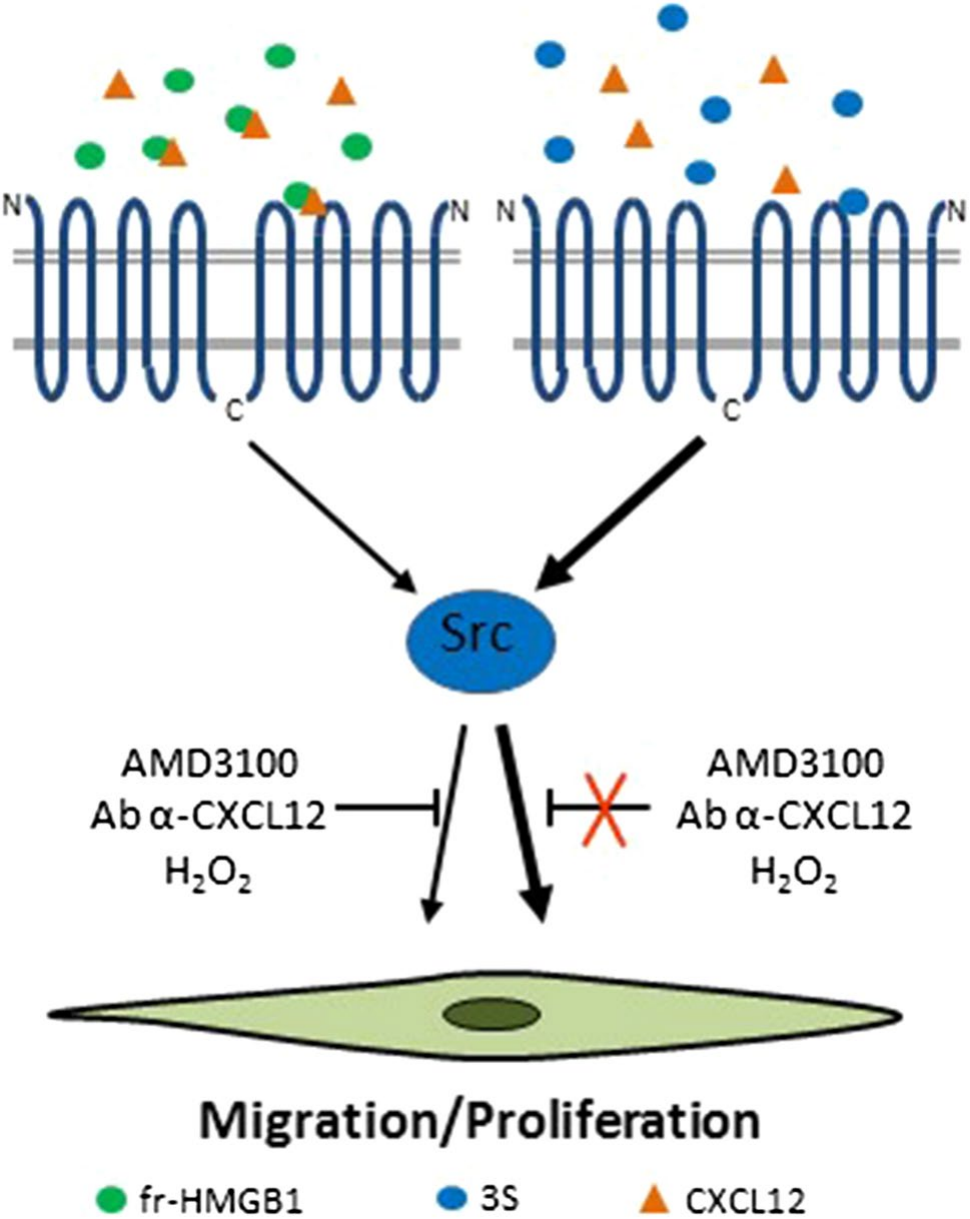
The non-oxidizable 3S mutant interacts directly with CXCR4. Fr-HMGB1 interacts with CXCL12 to promote cell migration and proliferation via CXCR4. A blocking antibody to CXCL12 or the CXCR4/CXCL12 inhibitor AMD3100 as well as the presence of H_2_O_2_ abolish fr-HMGB1 activities. On the contrary, 3S binds directly to CXCR4 in a CXCL12-independent manner and is more effective that fr-HMGB1 in inducing fibroblast migration mediated by Src activation and myoblast proliferation. It is likely that 3S recognizes a different site in the receptor compared to CXCL12, since neither AMD3100 nor an anti-CXCL12 antibody effectively block 3S-induced migration. Since 3S cannot be converted to oxidized HMGB1 forms, its chemotactic activity lasts in the presence of H_2_O_2_

CXCR4 is a receptor for SDF-1/CXCL12, an important chemotactic stimulus for leukocytes [76]. Fr-HMGB1 forms a heterocomplex with CXCL12 that protects CXCL12 from degradation and is responsible for CXCR4-mediated migration in mouse embryonic fibroblasts (MEFs), human cardiac fibroblasts (hcFbs), macrophages, dendritic cells, and myoblasts [13, 36, 53, 77] (Fig. 2). Fr-HMGB1 promotes muscle, skeletal, hematopoietic and liver regeneration through CXCR4, at least in part by recruiting tissue healing macrophages and promoting the transition of resident stem cells from the G_0_ to the G_alert_ phase, thereby accelerating their proliferation, migration and differentiation [13, 45] (Fig. 2). Interestingly, 3S interacts directly with CXCR4 with no need for CXCL12, and induces a conformational state of CXCR4 different from that triggered by CXCL12 [45, 53]. Accordingly, 3S exhibits an efficient Non-Receptor Tyrosine Kinase (Src)-mediated chemotactic activity even in the absence of CXCL12 [53]. The resistance to oxidative conditions and the direct binding to CXCR4 can explain the higher effectiveness of 3S relative to fr-HMGB1 [45, 53] (Fig. 3). Moreover, 3S binds inefficiently to TLR4/MD-2 and RAGE and does not exhibit pro-inflammatory properties [45, 53].

Hence, HMGB1 undergoes progressive redox modifications necessary to start, regulate and resolve the inflammatory response, but also to coordinate tissue repair and regeneration through the recognition of different receptors and interactors.

## HMGB1 in cardiac dysfunction

*Hmgb1* null mutations are lethal and mice die soon after birth with complex pleiotropic features, indicating that HMGB1 contributes to development and perinatal survival [17]. So far, there are no studies describing the mechanisms by which HMGB1 may affect proper heart development. On the other hand, HMGB1 seems to be dispensable for cellular homeostasis and proper organ function in the adult organism [18, 78]. In particular, mice with a cardiomyocyte-specific *Hmgb1* deletion do not show structural abnormalities or alterations in cardiac function and contractility and long-term survival [79]. Transgenic mice with cardiomyocyte-specific overexpression of HMGB1 (cHMGB1-Tg) display no significant differences in cardiac performances and plasma levels of HMGB1 in physiological conditions compared to the wild-type animals, however, after the induction of a cardiac damage they are partially protected from developing heart dysfunctions [80].

### Ischemic heart diseases

#### Myocardial infarction

Myocardial infarction (MI) is an ischemic insult resulting in loss of cardiomyocytes that are replaced by scar tissue [4]. Soon after MI, stressed cardiomyocytes release specific DAMPs that induce an acute and transient inflammatory response by activating PRRs [81]. Inflammatory cells clear debris from the infarcted area and secrete growth factors to activate myofibroblasts and vascular cells and initiate wound healing and tissue remodeling [4]. Finally, anti-inflammatory signals terminate leukocyte invasion and resolve inflammation, promoting tissue repair [4].

During MI, HMGB1 acts as a DAMP, modulates inflammation and functions as a regenerative factor. In a mouse model of MI induced by permanent coronary artery ligation, HMGB1 serum levels rapidly increase because of cardiac tissue necrosis. In the infarct zone HMGB1 expression peaks several days after MI: in the acute phase it is mainly localized in infiltrating inflammatory cells and later in CFs [82].

**Fig. 4:**
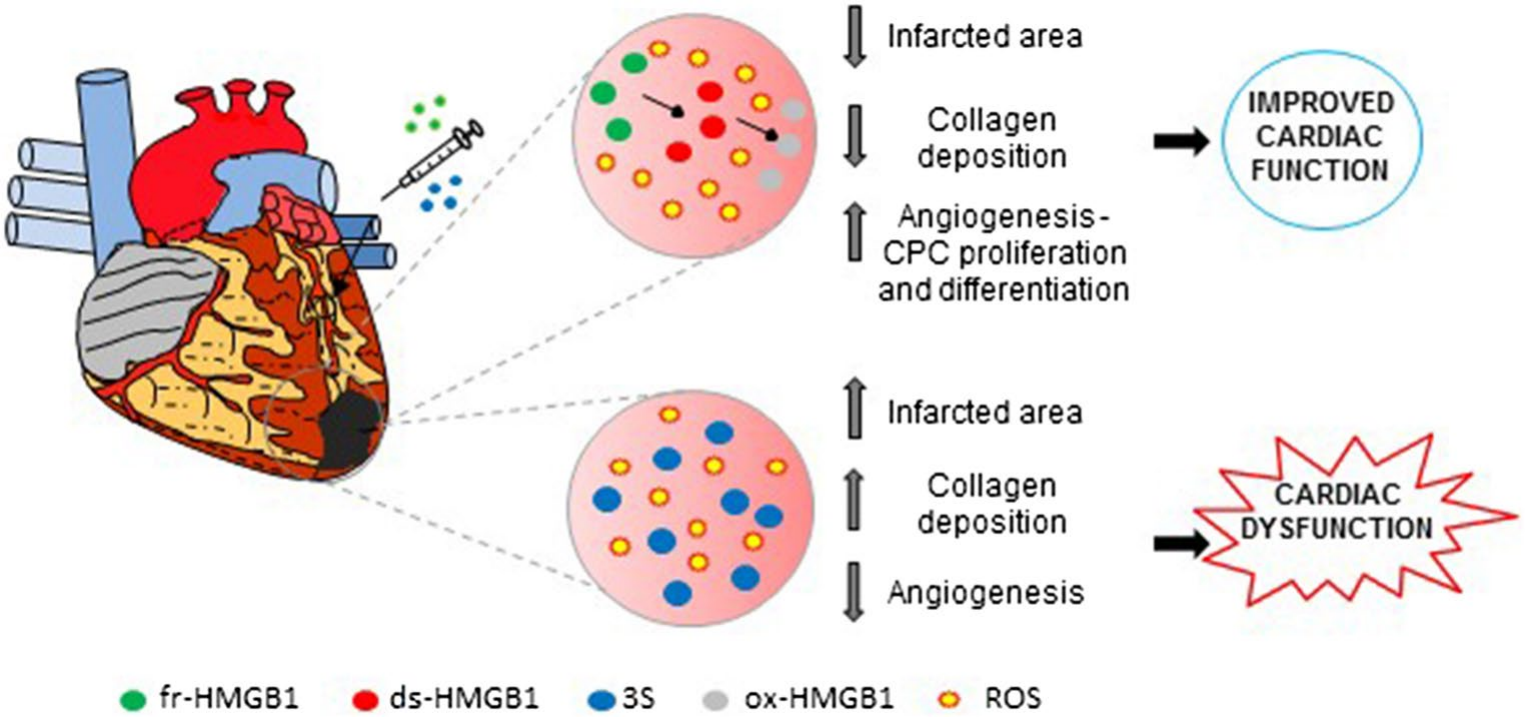
Fr-HMGB1 and 3S exert opposite effects in infarcted hearts. In an experimental model of myocardial infarction induced by permanent coronary ligation, fr-HMGB1 injection reduces the infarcted area and improves cardiac function because is able to promote angiogenesis and differentiation of resident cardiac stem cells (CPCs) into cardiomyocytes. The release of ROS subsequent to the infarction may progressively oxidize fr-HMGB1 to ds-HMGB1 and then to ox-HMGB1, which is important for the regenerative effect of HMGB1. On the contrary, the injection of the non-oxidizable 3S mutant reduces angiogenesis and causes an increase in the infarcted area and collagen deposition, leading to the worsening of cardiac dysfunction

Inhibition of extracellular HMGB1 after the infarct worsens cardiac dysfunction (Table 2). Indeed, injection of an anti-HMGB1 antibody 24 h post-infarction causes a reduction in inflammation and a marked infarct scar thinning [82]. Conversely, cHMGB1-Tg mice when undergoing infarction exhibit a smaller infarct size, preserved cardiac function and improved survival [80]. Infarcted cHMGB1-Tg animals show enhanced angiogenesis induced by increased mobilization and migration of bone marrow cells to the heart, their differentiation into endothelial progenitor cells and subsequent engraftment as vascular endothelial cells in new capillaries and arterioles [80, 83]. Similarly, mice injected with fr-HMGB1 in the ventricular tissue bordering the viable myocardium after MI exhibit improved Left Ventricular (LV) function due to neo-angiogenesis and a partial repopulation of the LV wall by newly formed cardiomyocytes derived from resident cardiac stem cells (CPCs; Fig. 4) [44, 53]. HMGB1 also attenuates cardiomyocyte apoptosis and stimulates their survival by inducing cell autophagy through AMP-activated protein kinase (AMPK) activation and inhibition of mammalian target of rapamycin complex 1 m (TORC1) [84]. Transcriptomic analysis confirmed that fr-HMGB1 enhances the expression of genes involved in endothelial cell migration and proliferation, stem cell differentiation and cardiomyocyte contraction [85]. HMGB1 also activates Translocation-Associated Notch Protein TAN-1 (Notch1) in the cardiomyocytes and increases the number and cardiomyogenic differentiation of CPCs [85]. HMGB1 influences CPC behavior in a paracrine manner as well, since conditioned medium from HMGB1-treated CFs induces CPC proliferation, migration and differentiation into endothelial cells [44, 86].

**Table 2:** Use of HMGB1 forms and antagonist in experimental models of cardiac disease

Experimental disease model	HMGB1 (redox form)	HMGB1 antagonist	Route of administration	Treatment effect		References
MI/rat	–	10 mg/Kg/day Poly anti-HMGB1	Subcutaneously 24 h after MI for 7 days	–	Detrimental—expansions of infarct scar and reduced inflammation	[78]
MI/cHMGB1-Tg mouse	Cardiomyocytes overexpression of HMGB1	–	–	Beneficial—reduced remodeling and increased angiogenesis	–	[79, 80]
MI/mouse	200 ng HMGB1 (fr-HMGB1)	–	Injections in the peri-infarcted area 4 h after MI	Beneficial—reduced remodeling and enhanced cardiac and vascular regeneration	–	[44, 53, 81, 82]
MI/mouse	200 ng non-oxidable 3S mutant	–	Injections in the peri-infarcted area 4 h after MI	Detrimental—increased collagen deposition and decreased angiogenesis	–	[53]
MI-Chronic HF/mouse	200 ng wtHMGB1 (fr-HMGB1)	–	Injections in the peri-infarcted area 2 weeks after MI	Beneficial—reduced remodeling and enhanced cardiac and vascular regeneration	–	[85]
MI-Chronic HF/rat	2.5 μg HMGB1	–	3 weeks after MI in the peri-infarcted area	Beneficial—reduced fibrosis	–	[84]
I/R mouse; 30 min occlusion-48 h reperfusion	10 μg HMGB1	400 μg BoxA	i.p. 1 h before I/R	Detrimental—enhanced fibrosis and inflammation	Protective—reduced necrosis, fibrosis and inflammation	[38]
I/R mouse; 30 min occlusion-24 h reperfusion	600 μg HMGB1	300 μg BoxA	i.p. 1 h before I/R	No effect	Protective—reduced necrosis and inflammation	[69]
I/R mouse; 30 min occlusion-24 h reperfusion	–	200 μg Poly anti-HMGB1; 70 μg mAb anti-TLR2	i.p. 1 h before I/R	–	Protective—reduced cardiac necrosis and apoptosis	[68]
I/R rat; 30 min occlusion-1 h reperfusion	–	mAb anti-HMGB1	Intravenous before reperfusion	–	Detrimental—increased cardiac necrosis, inflammation and infarct size	[88]
I/R rat; 30 min occlusion-4 h reperfusion	200 μg/Kg HMGB1	–	i.p. 24 h before I/R	Protective—reduced infarct size and inflammation	–	[100]
TAC/cHMGB1-Tg mice	Cardiomyocytes overexpression of HMGB1	–	–	Protective—reduced LV dysfunction, expression of hypertrophic markers and oxidative DNA damage	–	[107]
TAC/mouse	200 ng HMGB1	200 ng BoxA	Cardiac injection before ligation	Detrimental—increased cardiac hypertrophy and HF/	Protective—reversion of cardiac hypertrophy	[106]
Single dose of Dox i.p. 10 mg/kg/mouse	–	20 mg/kg BoxA	i.p. for 5 days starting 4 h after the Dox treatment	–	Protective—reduced apoptosis	[72]
Single dose of Dox i.p.17.5 mg/kg/HMGB1-Tg mouse	Cardiomyocytes overexpression of HMGB1	–	–	Protective—reduced apoptosis, LV dilation and remodeling	–	[111]
CMD induced by streptozocin/mouse	–	300 μg BoxA	Daily i.p. for 5 weeks after onset of hyperglycemia	–	Protective—reduced cardiac fibrosis and inflammation	[116]
I/R in CMD induced by streptozocin/mouse	–	400 μg BoxA	Daily i.p. starting 1 h before I/R for 5 days after onset of hyperglycemia	–	Protective—reduced cardiac fibrosis and inflammation and infarct size	[113]
ISO-induced cardiac fibrosis	–	10 mg/kg Gly	Daily i.p. for 15 days 1 h before ISO injection	–	Protective—reduced cardiac fibrosis	[119]
EAM (MyHC)/mouse	–	400 μg mAb anti-HMGB1	i.p. daily	–	Protective—reduced inflammation and fibrosis	[122]
EAM (TnI)/mouse	–	10 mg/Kg Gly or 50 μg mAb 2G7 anti-HMGB1	i.p. daily	–	Protective—reduced cardiac inflammation and dysfunction	[124]
LPS i.p. 10 mg/Kg/mouse	–	600 μg BoxA or 100 mg/Kg Gly	i.p. 4 h after LPS	–	Protective—reduced cardiac dysfunction	[127]

*cHMGB1-Tg* HMGB1 cardiac overexpression, *DCM* diabetic cardiomyopathy, *Dox* doxorubicin, *DNA* deoxyribonucleic acid, *EAM* experimental autoimmune myocarditis, *fr-HMGB1* fully reduced HMGB1, *Gly* glycyrrhizin; *HF* heart failure, *I/R* ischemia/reperfusion, *ISO* isoproterenol, *LPS* lipopolysaccharide, *LV* left ventricular, *mAb* monoclonal antibody, *MI* myocardial infarction, *MyHC* cardiac myosin heavy chain, *Poly* polyclonal antibody, *TAC* transverse aortic constriction, *TLR* toll-like receptor, *wtHMGB1* wild type HMGB1

Notably, the oxidizing environment generated after MI affects HMGB1 activities, since the injection of the non-oxidizable 3S mutant in infarcted mice worsens cardiac performance and enhances collagen deposition by increasing the number of myofibroblasts. This is possibly to a direct and sustained over-activation of CXCR4 (Fig. 4), as 3S is active at lower concentrations relative to fr-HMGB1 and in oxidizing conditions in stimulating hcFb migration and Src phosphorylation [53] (Fig. 3).

HMGB1 treatment improves cardiac recovery also in the context of post-MI chronic failing heart by attenuating inflammation in the peri-infarcted area and reducing LV remodeling and fibrosis [87, 88]. In this context, HMGB1 lowers collagen deposition by directly affecting Matrix Metallopeptidase 9 (MMP9) activity and Metalloproteinase Inhibitor 3 (TIMP3) expression through the induction of the microRNA (miR)-206 in hypoxic CFs [88].

Hence, in both acute and chronic MI higher levels of cardiac HMGB1 or exogenous administration of fr-HMGB1 elicits protective effects by modulating inflammation, enhancing cardiomyocytes regeneration and angiogenesis, and reducing fibrosis (Table 2). Interestingly, the progressive oxidation of fr-HMGB1 seems important to orchestrate correctly tissue healing after infarction. The involvement of HMGB1 receptors in MI has not been studied yet.

#### Ischemia/reperfusion (I/R)

In ischemic tissues, the decrease in blood supply leads to a deficit in oxygen and nutrients. In the heart, if ischemia is prolonged, cardiomyocytes die of necrosis and apoptosis. Reperfusion restores blood flow and can mitigate some of the deleterious effects of ischemia, but at the same time, is responsible for the massive leukocytes recruitment and the excessive production of free oxygen radicals at the injury site, which can cause additional damage exceeding the initial ischemic injury [89].

Several studies report an increase of circulating as well as myocardial HMGB1 levels in experimental models of I/R [38, 90, 91]. Circulating HMGB1 derives from necrotic cardiomyocytes and active secretion by hypoxic cardiac and infiltrating inflammatory cells [38, 68]. Myocardial HMGB1 expression increases soon after ischemia and remains high several days after reperfusion [38, 92]. Upregulation and release of HMGB1 has been described also in isolated neonatal murine cardiomyocytes (NMCMs) after hypoxia/reoxygenation (H/R) [38, 68, 92]. Extracellular HMGB1 alone or in concert with Tumor Necrosis Factor-α (TNFα) enhances H/R-induced cardiomyocyte apoptosis through the activation of Jun N-terminal kinase (JNK) and NF-κB via TLR2/4. Recently, Tian et al. described a signaling axis involving cardiac HMGB1 and splenic RAGE showing that circulating HMGB1 released from necrotic cardiac tissue after prolonged ischemic insult activates splenic leucocytes through RAGE to produce neutrophils that migrate to the injured myocardium [93]. This contributes to infarct exacerbation during reperfusion [93]. Oxidative and nitrosative stress control HMGB1 expression and release during I/R [90, 94]. The 5,10,15,20-tetrakis(2,4,6-trimethyl-3,5-sulfonatophenyl)porphyrinato iron III (FeTPPS), a selective peroxynitrite scavenger, significantly reduces myocardial HMGB1 expression and tissue injury triggered by I/R [90]. Similarly, pharmacological or herbal compounds, like geranylgeranylacetone, osthole, isoproterenol, asperosaponin, astilbin, minocycline, quercetin, and celastrol prevent HMGB1 upregulation and exert a protective effect [95–102].

**Fig. 5:**
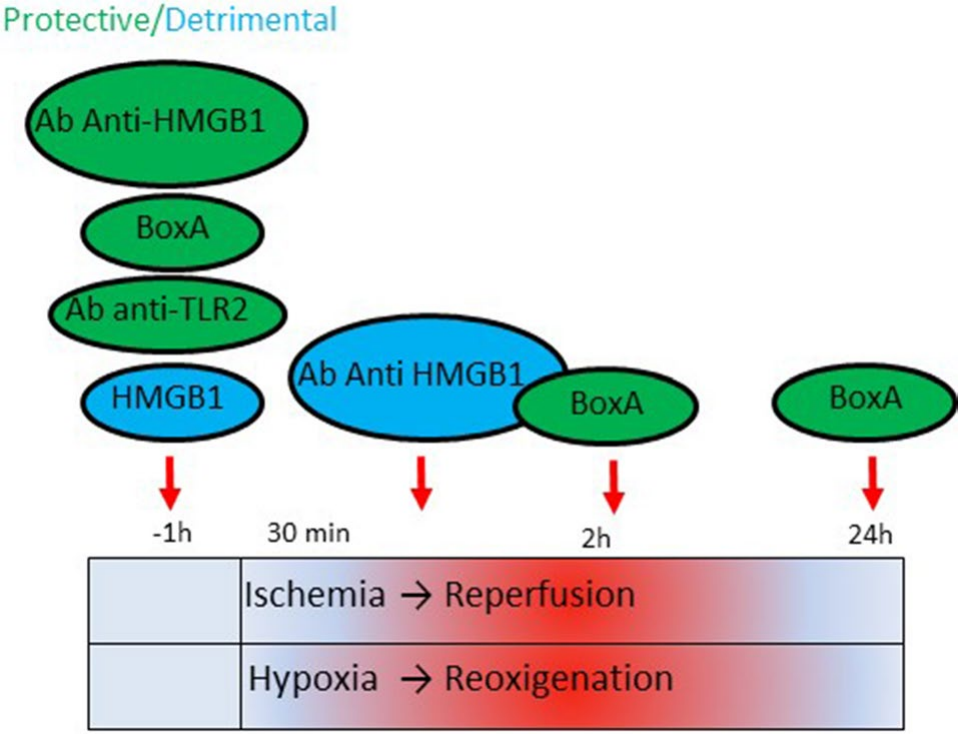
Effect of HMGB1 blocking during cardiac ischemia/reperfusion (I/R) damage. The cartoon indicates the protective or the detrimental consequences of administering HMGB1 antagonists or recombinant HMGB1 protein at different timing—pre-ischemic or post-ischemic phase—during I/R

Several HMGB1 inhibitors have been tested during I/R; however, the timing (pre-ischemic or post-ischemic phase), the dose or the mode of their administration appeared crucial in determining the final effect (Table 2; Fig. 5). Blocking HMGB1 before I/R exerts a protective action [38, 69, 103]. Injection of BoxA, an inhibitory domain of HMGB1, 1 h before I/R injury improves recovery of heart function by reducing cardiac necrosis, infarct size and inflammation [38]. Conversely, pre-treatment with high doses of recombinant HMGB1 is detrimental [38]. The pro-inflammatory activity of HMGB1 depends on RAGE/MAPKs/NF-κB and TLR2 activation since *Rage*^−^*/*^−^ and *Tlr2*^−^*/*^−^ mice are protected from I/R injury even after administration of HMGB1 [38, 69]. The two receptors are likely to cooperate in these experimental settings.

The N-terminal lectin-like domain (LLD) of thrombomodulin is known to sequester HMGB1 and prevent receptor binding [68, 104]. Mice lacking LLD showed elevated levels of HMGB1 expression in cardiac cells after I/R and developed larger infarcts due to massive cardiac inflammation and apoptosis. This phenotype is reverted with antibodies against HMGB1 or TLR2, or soluble LLD injected before I/R [68].

Intravenous administration of an anti-HMGB1 antibody in the post-ischemic phase and before reperfusion in rats determines an enlargement of the infarct size and increased levels of TNFα and the inducible nitric oxide synthase (iNOS) [91]. Accordingly, HMGB1 treatment after global I/R injury decreases infarct size and levels of myocardial pro-inflammatory factors [105]. Finally, treatment with BoxA after reperfusion exerts a protective action attenuating myocytes apoptosis [92] (Table 2; Fig. 5).

Thus, the effect of HMGB1 in I/R is highly context-specific. It is likely that the redox state of the protein plays a significant role and further studies are worth.

### Cardiac hypertrophy

Cardiac hypertrophy, the progressive thickening of cardiac muscle caused by cardiomyocyte hypertrophy, is the adaptive response of the heart to various stressors, including pressure or volume overload and MI [106].

In a mice model of pressure overload-induced cardiac hypertrophy by thoracic transverse aortic constriction (TAC), myocardial HMGB1 levels are upregulated because of infiltrating cells and higher expression in cardiomyocytes [107–109]. Neonatal rat cardiomyocytes (NRCM) subject to mechanical stress increase intracellular and extracellular HMGB1 in vitro [109] and this effect is abolished by fenofibrate, an inhibitor of cardiac hypertrophy [107]. Hypertrophic mediators like angiotensin II or Endothelin-1 induce acetylation and nuclear translocation of HMGB1 in NRCM; conversely, maintenance of stable intracellular HMGB1 levels prevent cardiac hypertrophy [110]. Accordingly, HMGB1-Tg mice undergoing TAC show higher survival and attenuated LV dilatation, systolic dysfunction, expression of hypertrophic markers and oxidative DNA damage (Table 2) [110].

In contrast, extracellular HMGB1 stimulates hypertrophy in isolated NRCMs, by increasing the cell surface and expression of Atrial Natriuretic Peptide through partial activation of calcineurin [108, 111]. Administration of BoxA in the heart reverses cardiac hypertrophy and delays heart failure (HF) induced by pressure overload, while recombinant HMGB1 aggravates it (Table 2) [109]. The ability of HMGB1 to induce cardiac hypertrophy does not involve RAGE, since *Rage*^−^*/*^−^ mice still develop cardiac hypertrophy after TAC [108].

Thus, HMGB1 may play dual functions in the context of cardiac hypertrophy depending on its subcellular localization. Preservation of intracellular HMGB1 levels prevents cardiac hypertrophy possibly by avoiding oxidative DNA stress, whereas extracellular HMGB1 promotes CM hypertrophy through an unknown receptor.

No published studies concerning the role of HMGB1 in hypertrophic obstructive cardiomyopathy are available so far. However, it is possible that the findings described above will be relevant to all conditions associated with an increase in myocardial mass, including hypertrophic obstructive cardiomyopathy.

### Anthracycline-induced cardiomyopathy

The survival rate of cancer patients has increased in the last years due to chemotherapy. However, many chemotherapeutic agents are cardiotoxic, eventually influencing the quality life of survivors [112]. Anthracyclines such as Doxorubicin (Dox) and Adriamycin (ADR) are widely used and effective anticancer chemotherapeutic agents associated with acute and late-stage dose-dependent cardiotoxicity. In the heart, these drugs lead to DNA damage, mitochondrial defective biogenesis and dysfunction that along with oxidative stress cause cardiac cells death and eventually HF [112]. Autophagy is critical for ADR-induced cardiotoxicity [113].

Administration of anthracyclines in mice increases the levels of cardiac and circulating HMGB1, likely released by necrotic cells [72, 113, 114]. Peroxynitrate species formation following Dox treatment regulates HMGB1 expression and release through JNK activation [72]. Injection of BoxA as well as genetic ablation of *Tlr4* reduce the cardio-depressive action of Dox by partially abrogating CM apoptosis (Table 2) [72]. ADR reduces the expression of the transcriptional activator Yes-associated protein (YAP) that, in turn, promotes HMGB1 upregulation in cardiomyocyte-like cells [113]. Silencing of HMGB1 reduces ADR-dependent autophagy and apoptosis by downregulating Light chain 3-phosphatidylethanolamine conjugate II (LC3II) and caspase-3, respectively [113].

Higher expression of cardiac HMGB1 exerts a protective effect in the pathogenesis of Dox-induced cardiomyopathy (Table 2). HMGB1-Tg mice treated with Dox show higher survival and attenuated LV dilatation and remodeling [114]. Higher levels of HMGB1 suppress mitochondrial vacuolization and dysfunction and cardiomyocyte apoptosis in response to Dox by upregulating heat shock protein beta 1 that, in turn, prevents caspase-3 activation [114].

### Diabetic cardiomyopathy (DCM)

MI is the most frequent cause of death in the diabetic population. HF is frequently associated with diabetes mellitus (DM) and, when it occurs independently of hypertension and coronary artery disease, is denominated diabetic cardiomyopathy (DCM). Long-term hyperglycemia triggers myocardial contractile dysfunction caused by cardiomyocyte hypertrophy, apoptosis and interstitial fibrosis [115].

CFs, CMs and macrophages cultured in hyperglycemic concentration of glucose (HG) exhibit enhanced HMGB1 expression [116, 117] and the protein is actively secreted from the nucleus to the extracellular space [117]. HG-dependent activation of the Phosphoinositide-3-kinase (PI3 K)/Protein-chinasi B (AKT) pathway is responsible for the upregulation of HMGB1 levels in CMs [118]. Silencing of HMGB1 or RAGE in these cells attenuates the NF-κB-mediated inflammatory cytokine production induced by HG [116]. Likewise, streptozotocin (STZ)-induced type 1 diabetes mellitus mice exhibit higher myocardial and circulating HMGB1 levels compared with control animals [40, 116, 119, 120]. Inhibition of RAGE or HMGB1, or BoxA administration in mice protects from (STZ)-induced cardiac dysfunction, reducing fibrosis and inflammation (Table 2) [40, 116, 119]. HMGB1 is required for HG-induced CMs apoptosis mediated by caspase-3 and BCL2 Associated X (Bax) activity via Extracellular signal–regulated kinase (ERK)1/2 activation [40]. HMGB1 also directly regulates CF proliferation and the profibrotic activity through MAPKs and AKT pathways [117] or through the reduction of the antifibrotic cytokine IL-33 via engagement of TLR4 [119, 121]. Recently, Wu et al. showed that interaction of HMGB1 with TLR2 alters the autophagy response in CFs causing an extensive synthesis of collagen I and α-smooth muscle actin (SMA) [122]. In vivo Glycyrrhizin (Gly) interferes with the HMGB1-TLR2 axis and alleviates cardiac fibrosis induced by isoproterenol (ISO) treatment [122]. The anti-oxidative properties of resveratrol reduced HMGB1 upregulation and signaling and ameliorated fibrosis and inflammation in diabetic hearts [120].

Diabetic animals subjected to I/R show an additional enhancement of HMGB1 cardiac and pro-inflammatory expression [95, 116]. Astilbin, a flavonoid compound that attenuates cardiac remodeling after I/R in diabetic rats, prevents HMGB1 upregulation [95]. Treatment of diabetic animals with BoxA early before I/R abrogates RAGE-dependent MAPKs and NF-κB activation and results in an improvement of cardiac contraction and diminished infarct size, inflammation and post-infarct remodeling (Table 2) [116].

### Myocarditis

Myocarditis refers to inflammation of the heart characterized by infiltration of inflammatory cells after viral, bacterial and protozoan infections [123]. Myocarditis is often an autoimmune reaction as well characterized by antibody-mediated myocardial damage [124]. For these reasons, myocarditis is the most common cause of acute HF and sudden death in young subjects [123, 124].

The role of HMGB1 in the pathogenesis of myocarditis has been evaluated for the first time in a mouse model of experimental autoimmune myocarditis (EAM) induced by inoculation of the cardiac myosin heavy chain (MyHC) peptide in the susceptible BALC/c mice strain [125]. This model replicates CD4+ T-cell-mediated autoimmune diseases and is characterized by myocardial necrosis, inflammatory infiltration and cardiac fibrosis. HMGB1 increased in cardiac tissue and in the blood of EAM mice and its blockage with an anti-HMGB1 antibody reduced infiltration of T helper-17 (Th17) cells, serum levels of inflammatory molecules and cardiac fibrosis [125]. HMGB1 directly affects Th17 cell polarization, differentiation and proliferation [125, 126]. More recently, Bangert et al. set up a murine model of cardiac Troponin I (TnI)-induced EAM [127]. HMGB1 inhibition with a specific antibody or glycyrrhizin decrease TnI-induced cardiac inflammation and dysfunction. Remarkably, HMGB1-Adeno-associated virus (AAV) mice, characterized by cardiomyocyte specific overexpression of HMGB1 encoded by an adeno-associated virus, exhibit basal cardiac inflammation, fibrosis and dysfunction that were further exacerbated after TnI immunization [127]. HMGB1 acts via a RAGE-independent mechanism [127] and TLR2 and/or TLR4 may be involved since an upregulation of both receptors is observed in these mice (Table 2) [127].

### Sepsis-induced cardiomyopathy

Cardiac dysfunction is a well-documented consequence of sepsis and septic shock [128]. Treatment of animals with LPS upregulates HMGB1 in cardiomyocytes and increases its levels in the bloodstream [129, 130] and both BoxA and glycyrrhizin ameliorate LPS-induced depression of cardiac contractility (Table 2) [130]. LPS promotes HMGB1 secretion via TLR4-dependent PI3 Kγ activation in isolated NMCMs and CFs/myofibroblasts [130]. Activation of PI3 K/AKT signaling, which prevents cardiac dysfunction during septic cardiomyopathy, is associated to inhibition of cardiac HMGB1 expression and translocation and of inflammation [131, 132].

### Idiopathic dilated cardiomyopathy

It is noteworthy that most animal models of dilated cardiomyopathy are related to a specific cause such as MI, aortic constriction, anthracyclines, myocarditis, and diabetes; the role of HMGB1 in these conditions has been addressed above. Idiopathic dilated cardiomyopathy is a clinical diagnosis by exclusion and no preclinical studies have examined HMGB1 in this condition in animal models of spontaneous cardiomyopathy.

### HMGB1 as a diagnostic and prognostic biomarker in human cardiac dysfunctions

#### Acute MI

Several clinical studies have reported elevated levels of circulating HMGB1 after acute MI [82, 133–138]. Goldstein et al. were the first to report that serum HMGB1 levels were significantly elevated in a small number of patients with myocardial ischemia compared to aged- and sex-matched healthy controls [136]. Later, Kohno et al. showed that circulating HMGB1 increased in patients with ST-elevation MI (STEMI) after undergoing percutaneous coronary intervention (PCI), peaking at 12 h after admission, and remaining elevated after 7 days compared to chronic stable angina (CSA) patients [82]. Peak HMGB1 levels independently associated with pump failure, cardiac rupture, in-hospital cardiac death and C-reactive protein (CRP) levels [82]. Furthermore, HMGB1 circulating levels positively correlated with plasma brain natriuretic peptide (BNP) determined 6 months after the infarction [82]. Accordingly, in a larger clinical study, Sørensen et al. reported elevated levels of plasma HMGB1 in a homogeneous group of STEMI patients after PCI when compared to control subjects [138]. Patients who died during the follow-up of 10 months exhibited higher levels of HMGB1 than surviving patients. Increased systemic HMGB1 levels, measured 3 h after hospital admission for STEMI, was also associated with reduced functional recovery after MI measured as heart rate recovery (HRR), peak oxygen consumption (VO_2peack_) and LV Ejection fraction (LVEF) determined 3–4 weeks after MI [134, 135].

Hashimoto et al. reported a strong association between serum levels of HMGB1 and adverse cardiovascular events after a median follow-up of 49 months in unstable angina (UA) and Non-STEMI (N-STEMI) Japanese patients with MI within 24 h of symptoms onset [137]. HMGB1 had a better ability to separate high- and low-risk subjects than CRP and the authors suggested it might be effective biomarkers for early risk stratification in UA and N-STEMI patients [137]. Finally, Andrassy et al. reported that circulating HMGB1 concentration, 2-4 days after hospital admission, represented a valuable negative prognostic mortality marker in acute coronary syndrome (ACS) patients, comparable to residual LV function, in STEMI and N-STEMI patients [133].

Thus, serum HMGB1 concentration early after the acute MI is predictive of the infarct size and risk of death, and represents a reliable prognostic biomarker for risk stratification and cardiovascular death in infarcted patients. Furthermore, the levels of HMGB1, days after the injury, may relate to the level of inflammation following the initial phase of tissue necrosis, and hence, may represent an early inflammatory prognostic indicator of HF development. The redox forms of HMGB1 released after cardiac injury have not been identified yet, thus further studies in this clinical setting are needed.

#### Chronic HF

Wang et al. show that elevated serum levels of HMGB1 are associated with systolic HF and worsening of LVEF caused by ischaemic or idiopathic dilated cardiomyopathy in both diabetic and non-diabetic groups of Asian patients [139]. Voltz et al. found that systemic levels of HMGB1 were upregulated also in Caucasian patients with severe HF, irrespective of the underlying cause of the disease (ischemic vs non-ischemic), compared to moderate/no symptoms subjects or healthy controls [140]. HMGB1 showed positive association with several markers and predictors of the disease such as N-terminal prohormone of brain natriuretic peptide (NTproBNP), creatinine levels, white blood cells (WBC) count and New York Heart Association (NYHA) classification, and was a predictor of all-cause death and heart transplantation [140]. Recently, Liu et al. examined serum HMGB1 amount in Chinese patients with chronic HF due to ischemic cardiomyopathy. HMGB1 levels were higher in HF subjects than in the control group and positively correlated with NTproBNP and NYHA functional class, while negatively correlated with LVEF [141]. Furthermore, HMGB1 was higher in patients who died than survivors after 12-month follow-up [141].

Hence, HMGB1 may be an alternative independent indicator of risk stratification in patients with chronic HF but further studies in more homogenous and larger population are needed.

#### Myocarditis

Bangert et al. found higher levels of HMGB1 in the plasma of a small number of patients diagnosed with acute myocarditis compared to patients without any signs of inflammation [127].

## Conclusion and future perspective

**Fig. 6:**
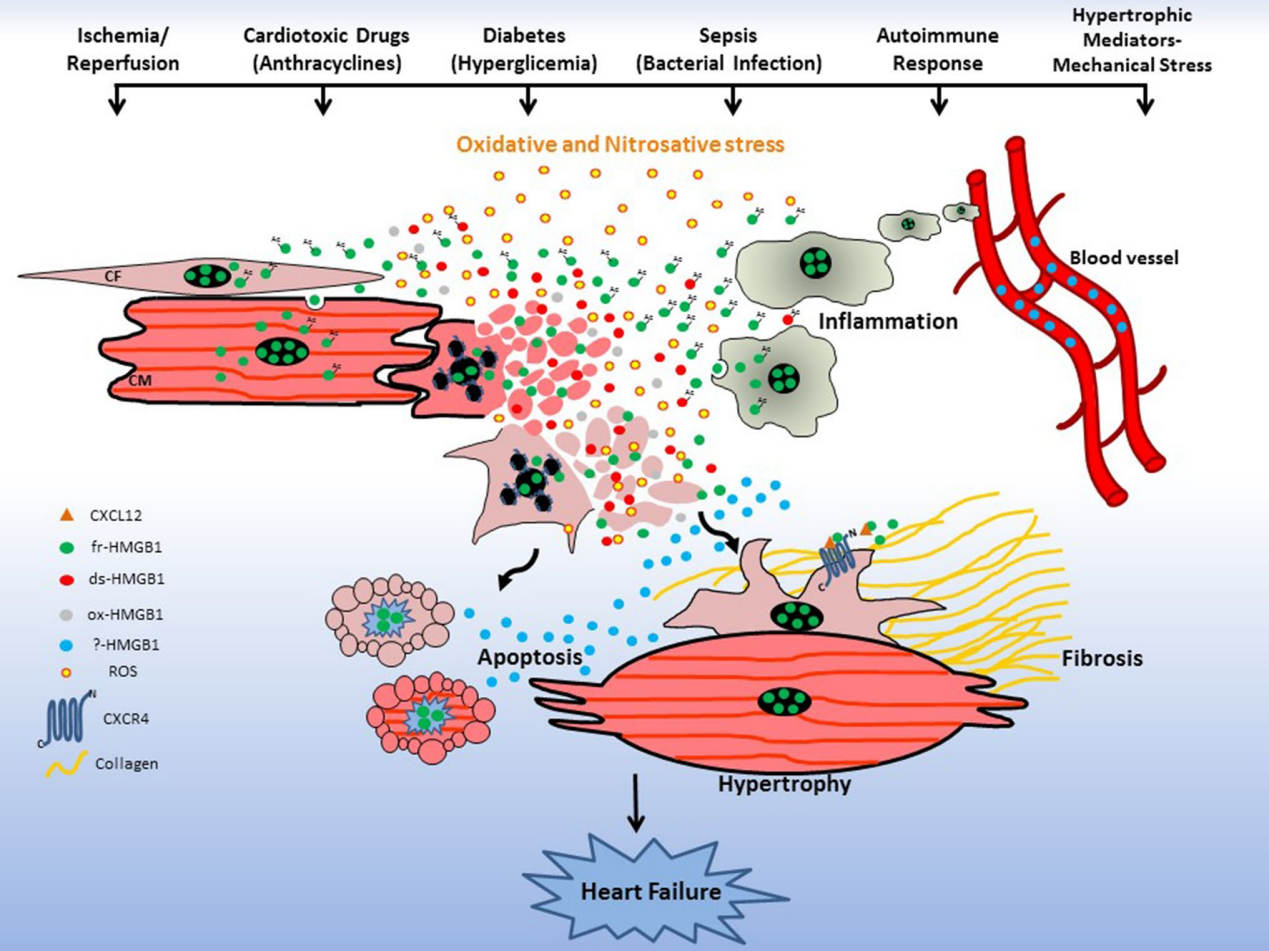
HMGB1 in cardiac dysfunctions: new perspective. Ischemia/reperfusion, cardiotoxic drugs, hyperglycemia, microbial infection, autoimmune responses or mechanical pressure produce oxidative and nitrosative stress that, in turn, induce tissue necrosis with consequent fr-HMGB1 passive release or acetylated fr-HMGB1 secretion from activated cardiac and recruited inflammatory cells. The extracellular fr-HMGB1 undergoes progressive oxidation and yet not specified redox forms (?) may exacerbate inflammation and induce cell apoptosis, cardiomyocytes (CM) hypertrophy and activation of cardiac fibroblasts (CF) to produce Collagen. These cell responses determine the development of cardiac hypertrophy and/or fibrosis and eventually heart failure. Modulation of the oxidative state of HMGB1 could be a strategy to limit inflammation and damage, and favor tissue repair. Furthermore, cardiac injuries lead to an increase in the blood levels of HMGB1 and the different modified forms of the protein may be associated with different disease stages, and could represent selective prognostic biomarkers of the extent of cardiac damage and help in risk stratification

HMGB1 is an ancestral chromatin-binding protein that during evolution has acquired an additional role as an extracellular “alarmin”. Very recent findings have demonstrated that extracellular functions of HMGB1 depend on its redox state [13, 45]. To date, the role of HMGB1 redox forms in the context of cardiac injuries remains mostly unexplored, opening promising new avenues of investigation in this field. Clearly, oxidative and nitrosative stress following an insult affects the expression, secretion and release of HMGB1 from cardiac cells. It is very likely that different levels of reactive oxygen species (ROS) production during cardiac damage regulates the kinetics of interconversion of fr-HMGB1 to ds-HMGB1 and ox-HMGB1 (Fig. 6), which could in part explain the discrepancies observed after administration of HMGB1 inhibitors in the various experimental models of cardiac injuries. For instance, I/R produces higher amounts of ROS than permanent ligation [142]. Moreover, it is not known which specific HMGB1 redox forms are involved in CMs contractility, hypertrophy and apoptosis, CFs activities or CPCs proliferation and differentiation (Fig. 6). Hence, the selective inhibition of HMGB1 redox forms or the modulation of HMGB1 oxidation could be a strategy to influence cardiac cell functions, limit inflammation and damage and favor heart repair. These are feasible approaches since selective inhibitors for HMGB1 redox forms have been identified (Table 1) and some ligands like Gly or heparin have been shown to modulate its oxidation kinetic [143].

Although extracellular HMGB1 is a risk factor for a number of heart diseases, applying neutralizing antibody or a biological inhibitor of HMGB1 as therapeutic agents is still confronted with a variety of challenges. In particular, several monoclonal antibodies against HMGB1 area available, and several are neutralizing. Some of these may hold clinical promise. However, they need to be optimized for clinical use, and clinical tests have not been initiated so far. BoxA is an effective inhibitor of HMGB1, but the molecule was described decade ago before the implication of HMGB1 in several pathologies was known, and cannot be patented; this alone makes clinical development very unlikely. Small molecules hold more promise, especially if they can be shown to act on specific redox of HMGB1. Glycyrrhizin is widely used in preclinical research, but has a number of drawbacks from the pharmacological point of view. Salicylic acid (SA) inhibits HMGB1, and this may contribute to the pharmacological properties of aspirin, of which SA is a metabolite. However, aspirin is not specific and efficacious enough for use in specific pathologies [15].

Another important point to be addressed concerns the identification of the redox forms of HMGB1 released after cardiac injury in clinical scenarios. Interestingly, specific HMGB1 redox forms appear more informative than total HMGB1 as clinical biomarkers [15, 144]. Likewise, modified HMGB1 forms may be also more informative than total HMGB1 as diagnostic and prognostic biomarker for risk stratification in the setting of acute cardiac insults such as myocardial infarction and myocarditis (Fig. 6). Taken together, these new observations may lead to the development of novel diagnostic and therapeutic strategies. Theoretically, the modified protein could be associated with different disease stages: non-acetylated and fr-HMGB1 could reflect the passive release of HMGB1 soon after injury and hence, the extent of the cardiac tissue loss, whereas acetylated and ds-HMGB1 could reflect active secretion, ongoing tissue inflammation and the presence of infiltrating immune cells.

Finally, how damaged cardiac cells can balance nuclear and extracellular functions of HMGB1 remains unexplored. Muscle regeneration is compromised in *Hmgb1*^+*/*−^ mice and skeletal healing after a fracture is impaired in *Hmgb1*^−*/*−^ mice [18, 45]. At the cellular levels, the dual location of HMGB1 seems to be functionally complementary; indeed, unstimulated *Hmgb1*^−*/*−^ macrophages have low histone content and activate genes associated with chemotaxis and inflammation similar to LPS-stimulated *Hmgb1*^+*/*+^ cells [14], suggesting that chromatin rearrangement caused by HMGB1-dependent nucleosome loss is an important epigenetic event in the cellular response to inflammation. Secretion of HMGB1 in the extracellular matrix implies a partial depletion of the nuclear level of HMGB1. Cells lacking HMGB1 contain fewer nucleosomes and more RNA transcripts genome-wide and have an increased susceptibility to DNA damage [6]. DNA damage by itself triggers HMGB1 translocation from nucleus to cytoplasm and this could further increase accumulation of DNA lesions leading to apoptosis. Various type of DNA damage and the activation of the DNA damage response (DDR) is observed in infarcted heart and pressure-overload HF [145]. Preliminary data indicate that maintenance of higher nuclear HMGB1 content protects CMs from apoptosis induced by Dox and detrimental hypertrophic stimuli by preventing DNA oxidative damage [114]. Thus, understanding how cells may preserve a proper level of nuclear HMGB1 to sustain efficient DNA repair may help to understand the progression of cardiac diseases.

In conclusions, further studies are necessary to understand the mechanisms by which extracellular and nuclear HMGB1 affects cardiac inflammation and repair/regeneration and how it can be manipulated to maximize its therapeutic potential in different forms of heart disease.
